# How Structure Determines Correlations in Neuronal Networks

**DOI:** 10.1371/journal.pcbi.1002059

**Published:** 2011-05-19

**Authors:** Volker Pernice, Benjamin Staude, Stefano Cardanobile, Stefan Rotter

**Affiliations:** 1 Bernstein Center Freiburg, Freiburg, Germany; 2 Computational Neuroscience Lab, Faculty of Biology, Albert-Ludwig University, Freiburg, Germany; Indiana University, United States of America

## Abstract

Networks are becoming a ubiquitous metaphor for the understanding of complex biological systems, spanning the range between molecular signalling pathways, neural networks in the brain, and interacting species in a food web. In many models, we face an intricate interplay between the topology of the network and the dynamics of the system, which is generally very hard to disentangle. A dynamical feature that has been subject of intense research in various fields are correlations between the noisy activity of nodes in a network. We consider a class of systems, where discrete signals are sent along the links of the network. Such systems are of particular relevance in neuroscience, because they provide models for networks of neurons that use action potentials for communication. We study correlations in dynamic networks with arbitrary topology, assuming linear pulse coupling. With our novel approach, we are able to understand in detail how specific structural motifs affect pairwise correlations. Based on a power series decomposition of the covariance matrix, we describe the conditions under which very indirect interactions will have a pronounced effect on correlations and population dynamics. In random networks, we find that indirect interactions may lead to a broad distribution of activation levels with low average but highly variable correlations. This phenomenon is even more pronounced in networks with distance dependent connectivity. In contrast, networks with highly connected hubs or patchy connections often exhibit strong average correlations. Our results are particularly relevant in view of new experimental techniques that enable the parallel recording of spiking activity from a large number of neurons, an appropriate interpretation of which is hampered by the currently limited understanding of structure-dynamics relations in complex networks.

## Author Summary

Many biological systems have been described as networks whose complex properties influence the behaviour of the system. Correlations of activity in such networks are of interest in a variety of fields, from gene-regulatory networks to neuroscience. Due to novel experimental techniques allowing the recording of the activity of many pairs of neurons and their importance with respect to the functional interpretation of spike data, spike train correlations in neural networks have recently attracted a considerable amount of attention. Although origin and function of these correlations is not known in detail, they are believed to have a fundamental influence on information processing and learning. We present a detailed explanation of how recurrent connectivity induces correlations in local neural networks and how structural features affect their size and distribution. We examine under which conditions network characteristics like distance dependent connectivity, hubs or patches markedly influence correlations and population signals.

## Introduction

Analysis of networks of interacting elements has become a tool for system analysis in many areas of biology, including the study of interacting species [1], cell dynamics [2] and the brain [3]. A fundamental question is how the dynamics, and eventually the function, of the system as a whole depends on the characteristics of the underlying network. A specific aspect of dynamics that has been linked to structure are fluctuations in the activity and their correlations in noisy systems. This work deals with neuronal networks, but other examples include gene-regulatory networks [4], where noise propagating through the network leads to correlations [5], and different network structures have important influence on dynamics by providing feedback loops [6], [7].

The connection between correlations and structure is of special interest in neuroscience. First, correlations between neural spike trains are believed to play an important role in information processing [8], [9] and learning [10]. Second, the structure of neural networks, encoded by synaptic connections between neurons, is exceedingly complex. Experimental findings show that synaptic architecture is intricate and structured on a fine scale [11], [12]. Nonrandom features are induced by neuron morphology, for example distance dependent connectivity [13], [14], or specific connectivity rules depending on neuron types [15], [16]. A number of novel techniques promise to supply further details on local connectivity [17],[18]. Measured spike activity of neurons in such networks shows, despite high irregularity, significant correlations. Recent technical advances like multiple tetrode recordings [19], multielectrode arrays [20]–[22] or calcium imaging techniques [23], [24] allow the measurement of correlations between the activity of an increasingly large number of neuron pairs in vivo. This makes it possible to study the dynamics of large networks in detail.

Since recurrent connections represent a substantial part of connectivity, it has been proposed that correlations originate to a large degree in the convergence and divergence of direct connectivity and common input [8] and must therefore strongly depend on connectivity patterns [25]. Experimental studies found evidence for this thesis in a predominantly feed-forward circuit [26]. In another study, only relatively small correlations were detected [27] and weak common input effects or a mechanism of active decorrelation were postulated.

In recent theoretical work recurrent effects have been found to be an important factor in correlation dynamics and can account for decorrelation [20], [22]. Several theoretical studies have analysed the effects of correlations on neuron response [28], [29] and the transmission of correlations [30]–[34], also through several layers [35]. However, the description of the interaction of recurrent connectivity, correlations and neuron dynamics in a self-consistent theory has not been presented yet. Even in the case of networks of strongly simplified neuron models like integrate and fire or binary neurons, nonlinear effects prohibit the evaluation of effects of complex connectivity patterns.

**Figure 1:**
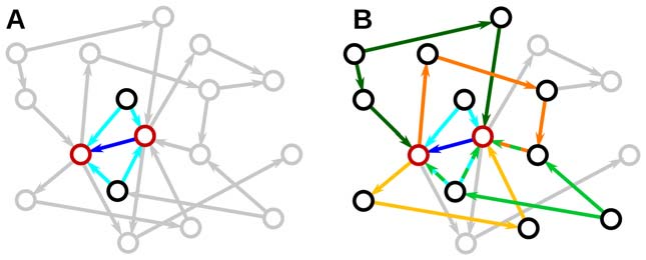
Connectivity induces correlations. **A**: Activity in a pair of neurons (red) in a network can become correlated due to direct connections (blue) and different types of shared input (cyan). **B**: For a complete description a large number of indirect interactions (yellow,orange) and indirect common input contributions (green) have to be taken into account. However, not all nodes and connections contribute to correlations (grey).

In [36], [37] correlations in populations of neurons were studied in a linear model that accounted for recurrent feedback. With a similar model, the framework of interacting point processes developed by Hawkes [38], [39], we analyse effects of different connectivity patterns on pairwise correlations in strongly recurrent networks. Spike trains are modeled as stochastic processes with presynaptic spikes affecting postsynaptic firing rates in a linear manner. We describe a local network in a state of irregular activity, without modulations in external input. This allows the self-consistent analytical treatment of recurrent feedback and a transparent description of structural effects on pairwise correlations. One application is the disentanglement of the explicit contributions of recurrent input on correlations in spike trains in order to take into account not only effects of direct connections, but also indirect connectivity, see Figure 1.

We find that variations in synaptic topology can substantially influence correlations. We present several scenarios for characteristic network architectures, which show that different connectivity patterns affect correlations predominantly through their influence on statistics of indirect connections. An influential model for local neural populations is the random network model [40], [41], possibly with distance-dependent connectivity. In this case, the average correlations, and thereby the level of population fluctuations or noise, only depend on the average connectivity and not on the precise connectivity profile. The latter, however, influences higher order properties of the correlation distribution. This insensitivity to fine-tuning is due to the homogeneity of the connectivity of individual neurons in this type of networks. The effect has also been observed in a very recent study, where large-scale simulations were performed [42]. In networks with more complex structural elements, like hubs or patches, however, we find that also average correlations depend on details of the connectivity pattern.

Part of this work has been published in abstract form [43].

## Methods

### Recurrent networks of linearly interacting point processes

In order to study correlations in networks of spiking neurons with arbitrary connectivity we use the theory derived in [38], which we refer to as Hawkes model, for the calculation of stationary rates and correlations in networks of linearly interacting point processes. We only summarise the definitions and equations needed in the specific context here. A mathematically more rigorous description can be found in [38] and detailed applications in [44], [45].

We will use capital letters for matrices and lower case letters for matrix entries, for example 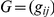. Vectors will not be marked explicitly, but their nature should be clear from the context. Fourier transformed quantities, discrete or continuous, will be denoted by 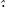, for example 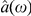. Used symbols are summarised in Table 1.

**Table 1:** Used symbols (in order of appearance).

Symbol	Description
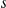	spike train vector
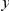	rate vector
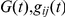	interaction kernel matrix, elements
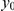	external input
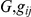	matrix of integrated kernels, elements
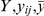	diagonal rate matrix, elements, average rate
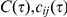	covariance density function matrix, elements
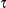	time lag
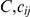	integrated covariance density matrix, elements
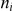	spike counts
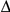	bin size
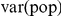	population count variance
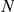	number of neurons
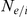	number of excitatory/inhibitory neurons
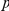	connection probability
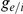	excitatory/inhibitory integrated interaction kernel
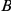 , 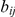	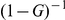 , elements
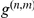	average correlation contribution of order 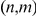
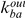	output connections from neuron type 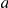 to 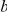
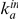	input connections to neuron type 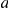
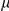	average interaction
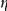	average common input
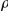	radius of bulk spectrum
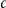	average correlation
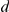	distance
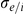	half width of boxcar-profile
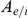	height of boxcar profile
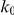	average out degree
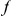	fraction hub to hub connections
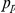	connection probability in patch
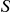	patch size

Our networks consists of 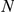 neurons with 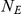 excitatory and 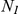 inhibitory neurons. Spike trains 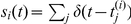 of neurons 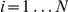 are modeled as realisations of Poisson processes with time-dependent rates 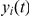. We have
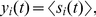
(1)


where 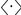 denotes the mathematical expectation, in this case across spike train realisations. Neurons thus fire randomly with a fluctuating rate which depends on presynaptic input. For the population of neurons we use the spike train vector 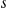 and the rate vector 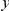. Spikes of neuron 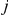 influence the rate of a connected neuron 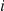 by inducing a transient rate change with a time course described by the interaction kernel 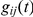, which can in principle be different for all connections. For the sake of simplicity we use the same interaction kernels for all neurons of a subpopulation. The rate change due to a spike of an excitatory presynaptic neuron is described by 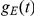 and of an inhibitory neuron by 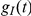. The total excitatory synaptic weight can then be defined as 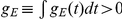 and the inhibitory weight accordingly as 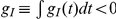. Connections between neurons are chosen randomly under varying restrictions, as explained in the following sections. For unconnected neurons 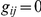. The evolution of the rate vector is governed by the matrix equation
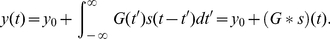
(2)


**Figure 2:**
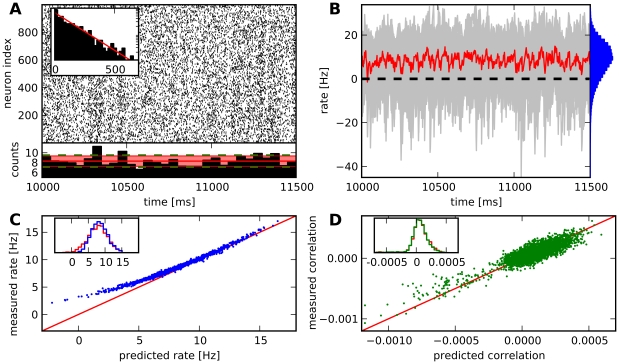
Hawkes' theory reproduces rates and correlations in a simulated random network. Network parameters are 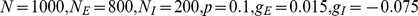. **A**: Top: spike raster plot showing asynchronous irregular activity (mean coefficient of variation 1.03). Inset: Inter-spike intervals of a typical spike train are exponentially distributed (logarithmic scale). Bottom: Population spike counts in bins of length 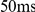. Mean 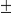 standard deviation (thick red line, shaded area), standard deviation from predicted correlations (green dashed line). **B**: Fluctuating rates of 50 neurons (grey traces), their average (red line) and distribution across time and neurons (blue). A small part reaches below zero (dashed line). **C**: Simulated time averaged rates scattered vs. predicted rates (blue). Diagonal (red) plotted for direct comparison. Inset: Distribution of predicted (red) and measured (blue) rates. Broad rate distribution with significant deviations from predictions only for small rates. **D**: Simulated correlations scattered vs. predicted ones. Larger errors due to finite simulation time. Inset: correlation distributions (green: measured, red: predicted). Although a non-vanishing part of fluctuating rates is below zero, most of the time averaged rates and correlations are predicted accurately.

The effect of presynaptic spikes at time 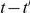 on postsynaptic rates is given by the interaction kernels in the matrix 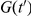 and depends on the elapsed time 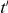. Due to the linearity of the convolution, effects of individual spikes are superimposed linearly. The constant spike probability 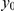 can be interpreted as constant external drive. We require all interactions to respect causality, that is 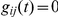 for 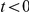. The Hawkes model was originally defined for positive interaction kernels. Inhibitory kernels can lead to negative values of 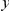 at certain times, so strictly one should use the rectified variable 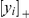 as a basis for spike generation. We assume further on that 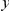 becomes negative only rarely and ignore the non-linearity introduced by this rectification. The effects of this approximation are illustrated in Figure 2. In the equilibrium state, where the expectation value for the rates 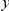 does not depend on time, we then have


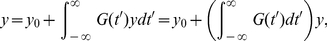
(3)

where we denoted the expectation 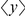 of the fluctuating rates by 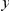 for notational simplicity. An explicit expression for the equilibrium average rates is
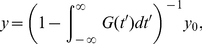
(4)


where 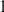 refers to the identity matrix.

We describe correlations between spike trains by the covariance density matrix 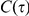. For point processes it is formally defined as the inverse Fourier transform of the spike cross-spectrum, but can in analogy to the case for discrete time be written as
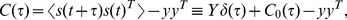
(5)


and corresponds to the probability of finding a spike after a time lag 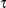, given that a spike happened at time 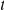, multiplied by the rate. The term 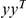 represents chance correlations such that for uncorrelated spike trains 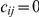 for 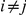. Due to the point process nature of spike trains, autocovariance densities 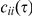 have a discontinuous contribution 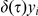. This discontinuity is separated explicitly from the continuous part 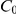 using the diagonal rate matrix 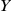 with the constant elements 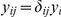 (here 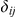 denotes the Kronecker delta). For independent spike trains 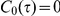 so that one recovers the autocorrelation density function of Poisson processes, 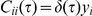. A self-consistent equation that determines the covariance density matrix is
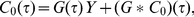
(6)


for 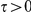. A key result in [38] is that, if the Fourier transform of the kernel matrix
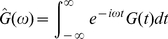
(7)


is known, (6) can be solved and the Fourier transform of the cross covariance density 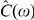 is given by
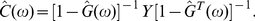
(8)


The definition of the Fourier transform implies that 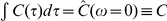 and accordingly 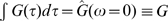, where we introduced the shortcuts 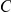 and 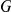 for the integrated covariance density matrix and kernel matrix, respectively. They are, from (8), related by
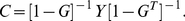
(9)


The rate Equation (4) becomes with these definitions
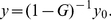
(10)


Equation (8) describes the time-dependent correlation functions of an ensemble of linearly interacting units. In this work we concentrate on purely structure-related phenomena under stationary conditions. Therefore we focus on the integrated covariance densities, which are described by Equation (9). Differences in the shape of the interaction kernels which do not alter the integral do not affect our results. One example is the effect of delays, which only shift interaction kernels in time. Furthermore we restrict ourselves to systems where all eigenvalues 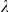 of 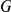 satisfy 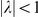. This condition guarantees the existence of the matrix inverse in (9) and (10). Moreover, if the real part 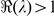 for any 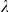, no stable equilibrium exists and network activity can explode. For further details see Section 1 of Supporting Text S1.

The matrix elements 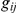 and 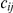 have an intuitive interpretation. The integrated impulse response 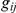 corresponds to the average number of additional spikes in neuron 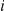 caused by an extra spike in neuron 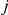.

The integrated cross-correlations 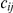, in the following simply denoted as correlations, equal, for asymptotically large counting windows 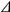, the covariances of spike counts 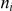 and 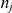 between spike trains 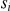 and 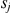,
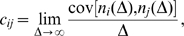
(11)


see for example [20], [46]. On the population level one finds for the population count variance normalised by the bin size, 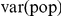, that
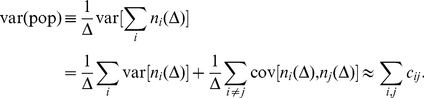
(12)


Strictly this is only true in the limit of infinitely large bin size. However, the approximation is good for counting windows that are large with respect to the temporal width of the interaction kernel. In this sense, the sum of the correlations is a measure for the fluctuations of population activity. Another measure for correlations that is widely used is the correlation coefficient, 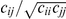. In this context it is not convenient, as the normalisation over the count variance destroys the simple relation to the population fluctuations. Even worse, as count variances are, just as covariances, influenced by network structure, for example global synchrony is not captured by this measure.

We simulated networks of linearly interacting point processes in order to illustrate the theory, Figure 2. In this network connections between all nodes are realised with constant probability 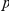. Parameters were chosen such that net recurrent input is inhibitory. The full connectivity matrix was used for the rate and correlation predictions in Equations (9) and (10) and the population count variance, Equation (12). Further simulation details are given below. This figure demonstrates that the approximation that fluctuating rates stay largely above zero gives good results even in effectively inhibitory networks with strong synapses. There are nonetheless slight deviations between prediction and simulation. On the one hand, fluctuations of the variable 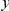 around a positive mean can reach below zero. This factor is especially relevant if rate fluctuations are high, for example because of strong synapses and low mean input. On the other hand, strongly inhibitory input can result into a negative mean value of 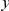 for some neurons. This can happen only for wide rate distributions and strong inhibition, since the ensemble average of 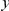 is always positive. In Figure 2C it is shown that only few neurons have predicted rates below zero, and that deviations between predicted and simulated rate distributions are significant primarily for low rates. The correlations in panel D are hardly affected. We found that for a wide range of parameters Hawkes' theory returns correct results for most of the rates and correlations even in effectively inhibitory networks.

### Simulation details

Simulations of linearly interacting point processes were conducted using the NEST simulator [47]. Spikes of each neuron were generated with a rate corresponding to the current value of the intrinsic variable 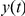. Negative values of 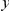 were permitted, but resulted in no spike output. Neurons received external drive corresponding to a constant rate of 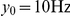. Incoming spikes resulted in an increase/decrease of 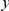 of amplitude 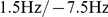 for excitatory/inhibitory spikes, which decayed with a time constant of 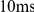. This corresponds to exponential interaction kernels with total weights 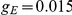 and 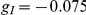. Synaptic delay was 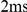. Simulation time step was 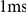 for the correlation and rate measurement and 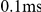 for spikes shown in the raster plot. In Figure 2 total simulation time was 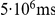. Data from an initial period of 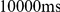 was dropped. Correlograms were recorded for the remaining time with a maximum time lag of 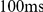 (data not shown). The value for the correlations was obtained from the total number of coincident spikes in this interval. The total number of spikes was used for the measurement of the rates, while population fluctuations were determined from 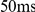 bins in the first 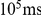.

## Results

### Powers of the connectivity matrix describe recurrent connectivity

In this section we address how recurrent connectivity affects rates and correlations. Mathematically, the kernel matrix 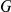 is the adjacency matrix of a weighted directed graph. Single neurons correspond to nodes and connections are weighted by the integrated interaction kernels.

With the shorthand
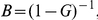



Equation (9) becomes
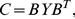
(13)


where the rates are given by (10), 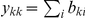. For simplicity we normalise the external input, 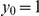. The matrix 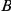 describes the effect of network topology on rates and correlations. Under the assumptions stated in the methods section, 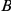 can be written as a geometric series,
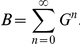



The terms of this series describe how the rates result from external and recurrent input. The matrix 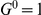 relates to the part of the rates resulting directly from external input. For 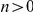, each of the single terms 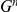 corresponds to indirect input of other nodes via paths of length 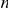. The element 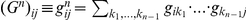 consists of the sum over all possible weighted paths from node 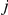 to node 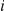 in 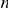 steps via the nodes 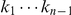 (note that 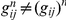). Since 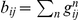, the elements of 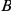 describe the influence of neuron 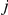 on neuron 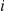 via all possible paths. Similarly
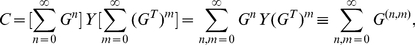
(14)


with 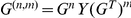. The first term 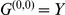 accounts for the integral of the autocorrelation functions of independent stationary Poisson processes, given by their rates. Higher-order terms in this series describe recurrent contributions to correlations and autocorrelation. The matrix elements of 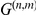 are
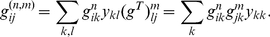
(15)


In these expressions, a term like 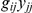 describes the direct effect of neuron 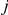 on 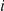, taking into account the interaction strength and the rate of the presynaptic neuron. For example, in the term with 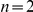 and 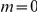 the elements 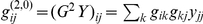 describe indirect input of 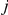 to 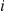 via all 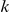. For 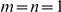, 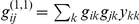 counts the common input of neurons 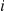 and 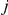 from all 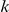. Altogether, the series expansion of the correlation equation describes how the full correlation between neurons 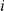 and 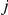 results from the contributions of all neurons 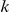, weighted by their rate, via all possible paths of length 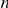 to node 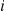 and length 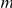 to node 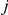, for all 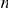 and 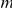.

**Figure 3:**
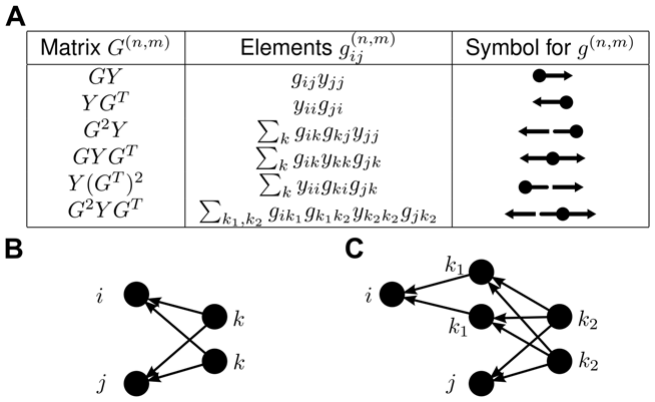
Correspondence of motifs and matrix powers. **A**: Examples for matrix expressions and symbols. **B**: Graphical interpretation of paths contributing to elements of the matrix 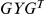. **C**: Same for the matrix 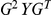.

These paths with two branches are the subgroup of network motifs that contribute to correlations. Further examples are given in Figure 3. The distribution of correlation coefficients depends on the distributions of these motifs. Note that larger motifs are built from smaller ones, hence distributions of different motifs are not independent.

As mentioned before, the sum (14) converges only if the magnitude of all eigenvalues of 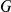 is smaller than one. This ensures that the feedback by recurrent connections does not cause runaway network activation. Both too strong recurrent excitation and too strong recurrent inhibition can lead to a divergence of the series. In such cases, our approach does not allow correlations to be traced back to specific network motifs.

Under this condition, the size of higher-order terms, that is the collective influence of paths of length 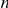 and 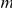, decreases with their total length or order 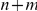. This can be stated more precisely if one uses as a measure for the contribution the operator norm 
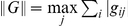
. After diagonalising 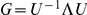 we have
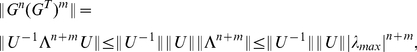
(16)


where 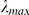 denotes the eigenvalue with the largest absolute value. If it is close to one, contributions decay slowly with order and many higher-order terms contribute to correlations. In this dynamic context the network can then be called strongly recurrent.

### Average correlations in regular networks do not depend on fine-scale structure

The average correlation across all pairs can be computed by counting the weighted paths between two given nodes. The average contribution of paths of length 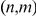 is
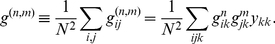
(17)


Let us separate the contributions from rates to the autocorrelations and define the average correlation 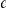 by
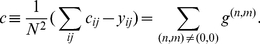
(18)


The population fluctuations are determined by 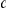,
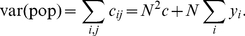
(19)


As a first approximation let us assume that every neuron in a given subpopulation 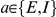 projects to a fixed number of neurons in each subpopulation 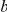, denoted by 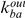. Furthermore, each neuron receives the same number of input connections from neurons of the two subpopulations, denoted by 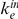 and 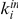. Synaptic partners are chosen randomly. These networks are called regular in graph theory, since the number of outgoing and incoming connections of each neuron, called the out- and in-degree, is identical for all neurons. This restriction can be relaxed to approximate certain types of networks, as we discuss in the respective sections. We set the external input 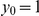. Then the total input to each neuron is 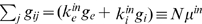. The shortcut 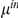 corresponds to the average input each neuron receives from a potential presynaptic neuron.

Since input is the same for all neurons, all rates are equal. Their value can be obtained as follows by the expansion of (10),
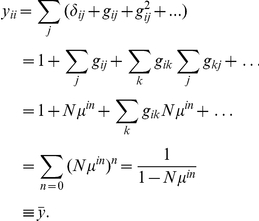



In a similar manner, analytical expressions for the average correlations can be obtained. Explicit calculations can be found in Section 2 of Supporting Text S1. In particular, the average correlation and hence the population fluctuations only depend on the parameters 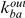 and 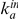.

Closed expressions can be derived in the special case where there is a uniform connection probability between all nodes, i.e.
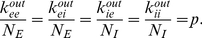
(20)


With 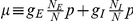 and 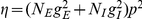 one finds for the individual contributions
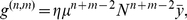
(21)


and the average correlation
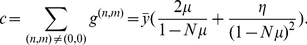
(22)


Here, 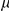 can be interpreted as the average direct interaction between two nodes and 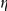 as the average common input shared by two nodes. Average correlations are determined by mean input and mean common input.

Equation (22) can be used as an approximation if the degree distribution is narrow. In particular this is the case in large random networks with independent connections, independent input and output and uniform connection probabilities. These conditions ensure that deviations from the fixed out- and in-degrees balance out on average in a large matrix. Numerical examples can be found in the following section.

### Random networks revisited

#### Indirect contributions of higher-order motifs decorrelate inhibitory networks

**Figure 4:**
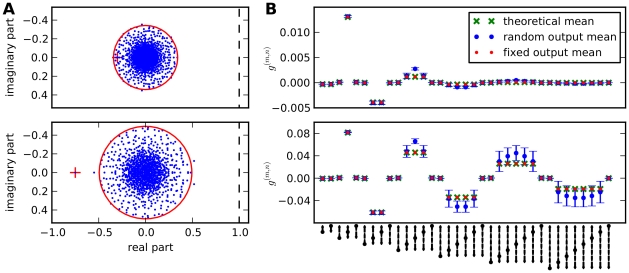
Motif contributions to average correlations in random networks. Top: low connectivity, 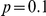. Bottom: higher connectivity, 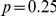. Other parameters as in Figure 2. **A**: Spectra of connectivity matrices (fixed out-degree), eigenvalues in the complex plane. Red circle: theoretical radius for bulk spectrum. Red cross: mean input to a neuron. The networks are inhibition dominated (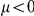) and real parts of all eigenvalues are below one (dashed line). **B**: Contributions of different motifs to average correlation. Comparison between theoretical prediction, random networks with uniform connection probabilities (average across 10 realisations, error bars indicate standard deviation), and networks with fixed out-degree. While in the sparse network only the first few orders contribute, higher orders contribute significantly in the dense network. The analytical expression for the average correlation reproduces the values for networks with fixed out-degrees and approximates the values for random networks. Within one order, chain motifs hardly add to correlations, while common input motifs have a larger contribution. Since inhibition dominates in the network, contributions are positive for even orders 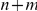 and negative for uneven orders. Refer to Figure 3 for the correspondence of symbols and paths.


In this section we analyse networks, where connections between all nodes are realised with uniform probability 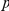. Using Equation (18) for the average correlation
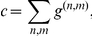



one can expand the average correlation into contributions corresponding to paths of different shapes and increasing length. In large random networks each node can connect to many other nodes. The node degree is then the sum of a large number of random variables, and the standard deviation of the degrees relative to their mean will be small. In this case, the constant degree assumption is justified, and Equation (21) gives a good approximation of the different motif contributions, see Figure 4. Decomposition of 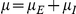 in an excitatory, 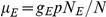, and inhibitory part, 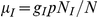, shows that terms of different length 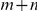 contribute with different signs in inhibition dominated networks (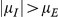):

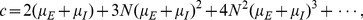
(23)

such that each term partly cancels the previous one. The importance of higher-order contributions can be estimated from the eigenvalue spectrum of the connectivity matrix. For large random networks of excitatory and inhibitory nodes, the spectrum consists of one single eigenvalue of the size 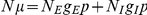 and a bulk spectrum of the remaining eigenvalues which is circular in the complex plane [48]. Its radius 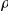 can be determined from
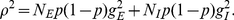
(24)


The value 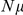 corresponds to the average input of a neuron, while 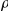 coincides with the input variance of a neuron. The effect of the connectivity on motif contributions and eigenvalue spectra is illustrated in Figure 4. A network is stable if neither the average recurrent input nor the input variance is too large, that is if 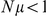 and 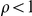. Random connectivity in neural networks can therefore, due the variability in input of different neurons, render a network unstable, despite of globally balanced excitation and inhibition (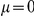) or even inhibition dominance.

#### Correlation distributions in random networks depend on connectivity

**Figure 5:**
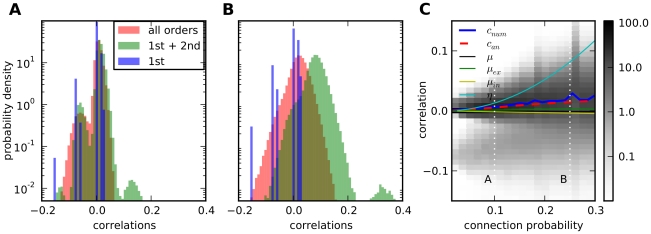
Strongly recurrent networks have broad correlation distributions. **A**: Low connectivity, 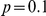, **B**: high connectivity, 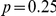. Other parameters as in Figure 2. The discrete distribution of direct interactions (blue) is washed out by second order terms (green) to a bimodal distribution for low and a unimodal distribution for high connectivity. higher-order terms (red) contribute significantly only for high connectivity. **C**: Correlation distributions change from a bimodal to a unimodal distribution for increasing connectivity (grey-scale indicates probability density). Average correlation (blue) increases smoothly and faster than the average interaction (black), which is the sum of excitatory (green) and inhibitory (yellow) interaction, but slower than the average common input (cyan) due to higher-order terms. The analytical prediction from Equation (22) for the average correlation (dashed red) fits the numerical calculation, especially for low connectivities. Vertical dotted lines indicate positions of the distributions in **A** and **B**.

By correlation distribution we denote the distribution of the entries 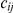 of the correlation matrix 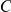. Its shape depends on the strength of recurrence in the network. Weak recurrence is characterised by 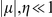, which is the case for low connectivity and/or small weights. In this case, mainly the first and second order terms in the expansion (14) corresponding to direct input, indirect input and common input contribute to correlations. For strongly recurrent networks longer paths contribute significantly and may change the distribution arising from lower order terms, compare Figure 5.

### Ring networks can have broad correlation distributions

Instead of purely random networks we now consider networks of 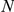 nodes arranged in a ring with distance dependent connectivity. The type of each neuron is determined randomly with probabilities 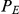 and 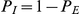, such that on average 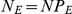 excitatory and 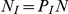 inhibitory neurons are distributed over the ring. Outbound connections of each neuron to a potential postsynaptic neuron are then determined from a probability profile 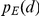 or 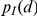, depending on the mutual geodesic distance 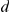 on the ring. The average interaction 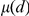 between two randomly picked neurons at a distance 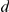 is
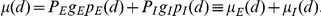



**Figure 6:**
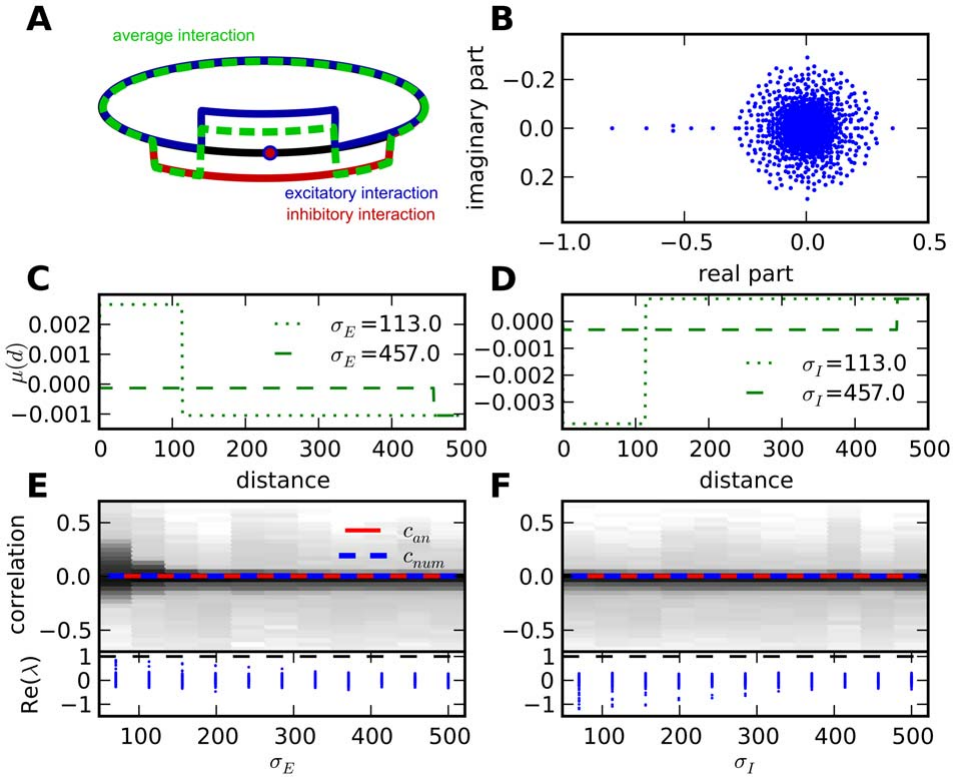
Correlation distributions depend on range of inhibition in ring networks. **A**: Distance dependent connectivity in a ring. Nodes are connected with a fixed weight to neurons with a probability depending on their mutual distance. Average interaction is the product of connection probability and weight averaged over populations. The connectivity profile may be different for excitatory neurons (red, positive weight) and inhibitory ones (green, negative weight). Average interaction on a randomly picked neuron at a distance corresponds to the sum (blue). **B**: Typical spectrum for a connectivity matrix with local inhibition. Parameters: 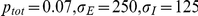, others as in Figure 2. **C,D**: Examples for average interaction profiles used in **E** and **F**. **C**: Global inhibition (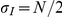) and local excitation, 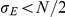 for small (dashed) and large (dotted) 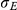, hat profile. **D**: Local inhibition and global excitation, inverted hat profile. Other parameters as in **B**. **E,F**: Top: Correlation distributions for fixed 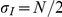 and increasing 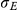 (**E**) and fixed 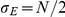 and increasing 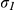 (**F**), logarithmic colour scale. Values between 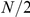 (random network) and 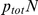 (connectivity in boxcar 0.5). Overall connectivity 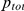 remains constant. Average correlation (dashed blue: numerical, red: analytical) does not change. Bottom: real parts of eigenvalues for corresponding connectivity matrices. Rings with local excitation tend to be less stable.

A sketch for this construction scheme is depicted in Figure 6A. For the connection probabilities we use a boxcar profile, 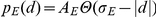 and 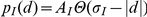, where 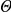 denotes the Heaviside step function. Neurons with a distance smaller than 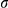 are connected with a probability 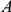, where 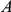 and 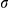 depend on the type of the presynaptic neuron.

The stability of such a network depends on the radius of the bulk spectrum. Additionally and in contrast to the random network, besides the eigenvalue corresponding to the mean input of a neuron, a number of additional real eigenvalues exist outside the bulk spectrum. A typical spectrum is plotted in Figure 6B. These eigenvalues are particularly pronounced for locally strongly connected rings with large 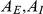 and belong to large scale oscillatory eigenmodes. The sign of these eigenvalues depends on the shape of the interaction profile. For short-range excitation and long-range inhibition (6C), that is a hat-like profile, these eigenvalues are positive and tend to destabilise the system. For the opposite, or inverted-hat case (6D), these eigenmodes do not affect stability, therefore stability is determined by the radius of the bulk spectrum. This can be seen as an analogue to the case of net inhibitory input in random networks.

As in a random network, the degree distribution of nodes in a ring network is narrow, hence Equation (22) is a good approximation for the average correlation if the total connection probability 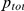 is independent on the neuron type,
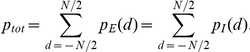



In this case the average correlation does not depend on the specific connectivity profile. However, the full distribution of correlations depends on the connection profile, Figure 6E and F. For localised excitation the eigenvalues of oscillatory modes get close to 1, rendering the network almost unstable, and many longer paths contribute to correlations. Since for ring networks neighbouring nodes can share a lot of indirect input, while more distant ones do not, this leads to more extreme values for pairwise correlations.

#### Correlations depend on distance

For distance dependent connectivity correlations are also expected to depend on the distance. We define the distance dependent correlation 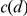 by
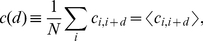
(25)


where 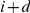 should be understood as 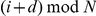 to reflect the ring structure, and the expectation 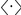 is taken over all nodes. Since this is also an averaged quantity, a similar calculation as in the case of the average correlation can be done. Since matrix products count the number of paths, one can show that expectation values of matrix products correspond to convolutions of the average interaction kernels 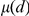. Details of the calculation can be found in Section 3 of Supporting Text S1. As before 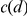 is expanded into terms corresponding to different path lengths,
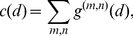
(26)


with 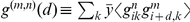, where 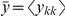 is the average rate. We note that
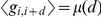



and define a distance dependent version of the average common input, 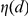, by
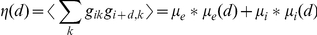



where 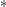 denotes discrete convolution. Using the discrete (spatial) Fourier transform,
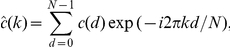



one finds for the single contributions
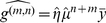
(27)


and for the complete correlations
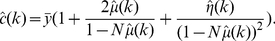
(28)


**Figure 7:**
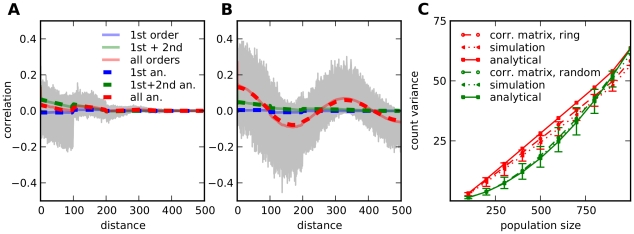
Distance dependence of correlations and population fluctuations. **A,B**: Evaluation of 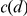 for parameters 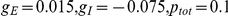 and 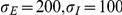, localised inhibition (**A**) and 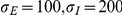, localised excitation (**B**). Other parameters as in Figure 2. Contributions of different paths, numerically (full lines) and analytically (dashed lines). Higher orders add up to extreme values for localised excitation but cancel out for localised inhibition. Correlations of individual neurons with distant neighbours vary considerably (grey, 50 traces shown). **C**: Variance of population spike counts over population size. Comparison between populations of neighbouring neurons in a ring and in a random network with fixed output. Plotted are results from analytical approximation, numerical calculation using the connectivity matrix and direct simulation, averaged over 5 populations in each case. Network Parameters: random network as in Figure 2, ring: 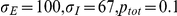. Simulation parameters: total simulation time: 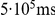, bin size for spike counts: 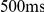, others as in Figure 2.

The discrete Fourier transform can be calculated numerically for any given connectivity profile. Results of Equations (27) and (28) are compared to the direct evaluation of (25) in Figure 7. The origin of the broad correlation distribution in Figure 6B can now be explained. For the hat-like profile, in a fixed distance, contributions of different order share the same sign and therefore add up to more extreme values. In an inverted hat profile, different orders of contributions change sign and cancel, leading to less extreme correlations and consequently a narrow distribution. The average correlation, however, is not affected.

#### Fluctuation level scales differently in ring and random networks

While the average correlation and therefore the variance of population activity in a network does not depend on structure in the networks considered so far, this is not true for smaller subnetworks. In ring-like structures, small populations of neighbouring neurons are more strongly correlated, and we expect larger fluctuations in their pooled activity. Generalising equation (12) slightly for a population 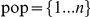 we define
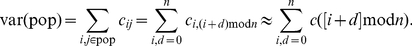
(29)


This expression can be evaluated numerically using Equation (28). For random networks, correlations do not depend on the distance. Hence the population variance increases quadratically with the number of elements. When increasing the population size in ring networks, more neurons which are further apart and only weakly correlated to most of the others are added, therefore a large part of their contribution consists of their rate variance and the population variance increases linearly. An example is shown in Figure 7. All curves approach the same value for a population size of 1000 (the complete population), but for smaller population sizes one finds the expected quadratic versus the linear dependency. If the members of the populations in a ring network are not neighbours, but randomly picked instead, the linear increase becomes quadratic, as in a random network (data not shown).

### Connected excitatory hubs of high degree or patches increase correlations

**Figure 8:**
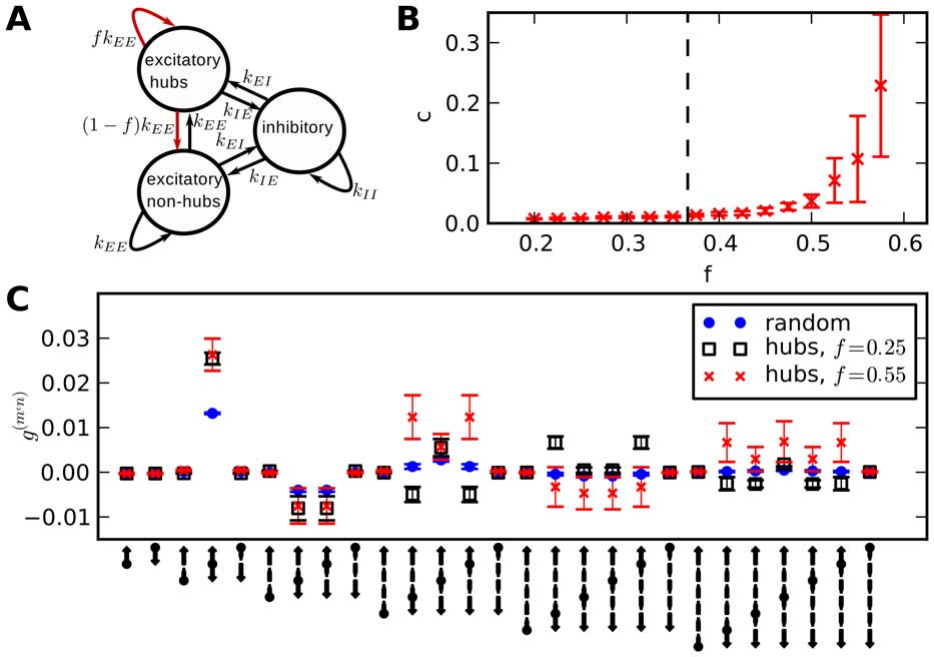
Higher-order contributions to correlations are increased by connected excitatory hubs. **A**: Construction. Excitatory neurons are divided into hubs (large out-degree) and non-hubs (small out-degree). The fraction of excitatory outputs from hubs to hubs 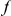 is varied. **B**: Average correlations increase with hub-interconnectivity 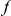 (average across 10 networks, error bars from standard deviation). Densely connected hubs (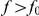, dashed vertical line) lead to large average correlations. **C**: Contribution to average correlations of different motifs for random network and networks with broad out-degree distribution and 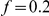 (disassortative network) and 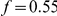 (assortative network). Average across 20 networks, error bars from standard deviation. Although contributions are different from random networks, in networks with broad degree distribution low order contributions (indirect and common input) are independent of hub interconnectivity, in contrast to certain higher-order contributions.

We found that in networks with narrow degree distributions average correlations are determined by global parameters like the population sizes 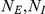 and overall connectivity 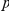, see Equation (22). In networks with broad degree distribution however, the regular-graph approximation is no longer valid. Thus, in such networks the fine structure of the connectivity will, in general, play a role in determining the average correlation. To elucidate this phenomenon, we use a network model characterised by a geometric degree distribution. The fine structure can then be manipulated without altering the overall connectivity. Specifically, the connection statistics of a given node will depend on the out-degree. The network model is defined as follows (compare Figure 8A). Out-degrees 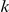 of excitatory and inhibitory neurons are chosen from a geometric distribution with a probability 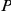



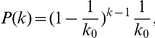


where the parameter 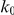 corresponds to the mean out-degree. The resulting distribution has a mean connection probability of 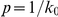 and a long tail. Excitatory neurons are then divided into classes according to their out-degree. We will call neurons with out-degree 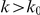 hubs and the rest non-hubs to distinguish the classes in this specific example. Postsynaptic neurons for non-hubs and inhibitory neurons are chosen randomly from all other neurons. For each hub we fix the fraction 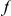 of connections that go to other hubs. The number of connections to excitatory neurons 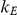 is chosen from a binomial distribution with parameter 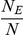. A number 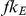 of the 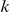 postsynaptic neurons are randomly chosen from other hubs, 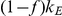 outputs go to non-hub excitatory neurons and 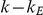 connections to randomly chosen inhibitory neurons. By varying 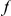 between 0 and 1, excitatory hubs can be chosen to form a more or less densely connected subnetwork. From the cumulative geometric distribution function, 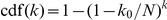, the expected fraction of hubs is 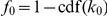, which is about 0.35 for 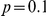. If 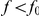 hubs are preferentially connected to non-hubs, otherwise hubs are more likely connected to each other.

By construction the parameters 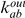 do not depend on 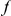. Hence terms with 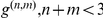, including common input, are also independent of 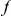. The statistics for longer paths are however different. If excitatory hubs preferentially connect to hubs, the number of long paths within the excitatory population increases. The effects on correlations are illustrated in Figure 8. Densely connected hubs increase average correlations. While the contributions of smaller motifs do not change significantly, from the larger motifs all but the pure chain motif contributions are affected.

**Figure 9:**
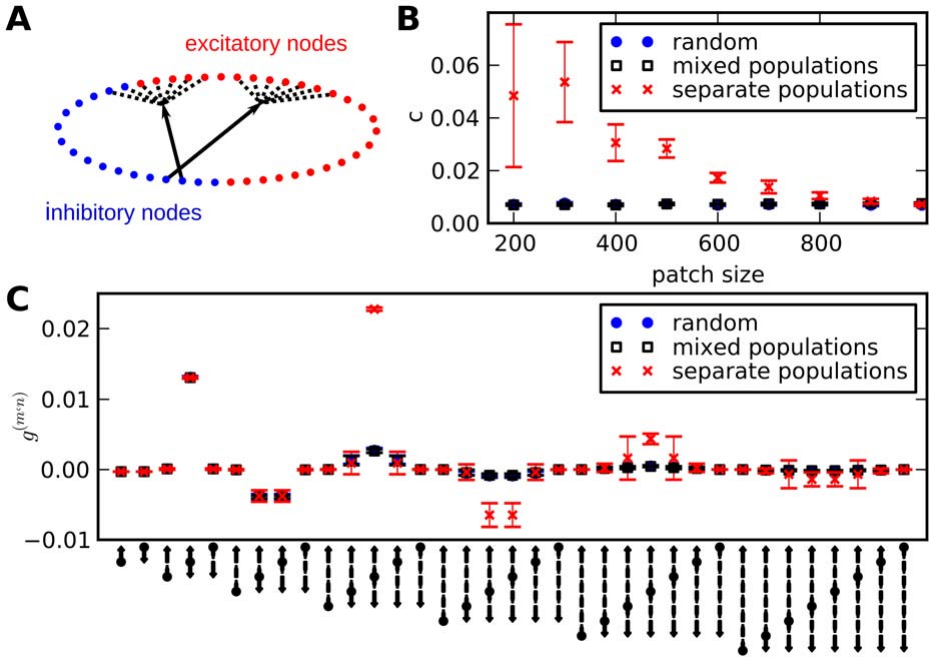
Patches in separate populations selectively affect high-order common input motifs. **A**: Connection rules for networks with patches. Output connections of a neuron are restricted to a randomly chosen region. **B**: Average correlations depending on patch size. Comparison between random networks, patchy networks with randomly distributed neuron type and separate populations, average across 5 networks, error bars from standard deviation. Only separate populations lead to increased correlations. Larger increase occurs for smaller patch-size. Total connection probability 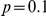, other parameters as in Figure 2. **C**: Contributions of different motifs. Differences to random networks occur only in common input terms of higher order. Patch size 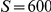.

Different effects can be observed in networks of neurons with patchy connections and non-homogeneous spatial distribution of neuron types. A simple network with patchy connections can be constructed from neurons arranged in a ring. We consider two variants: one where all inhibitory neurons are situated in the same area of the ring, compare Figure 9A, and one where they are randomly distributed over the ring. For each neuron, postsynaptic partners are chosen from a “patch”, a population of 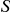 neighbouring neurons which is located at a random position, with a probability 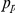. If neuron populations are not uniformly distributed, this leads to large variations in single neuron 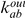, even if average values are kept fixed. We compare networks where excitatory and inhibitory neurons are spatially separate, Figure 9A, versus randomly mixed populations. In Figure 9B average correlations are compared to correlations in networks with random connectivity. If excitatory and inhibitory neurons are distributed randomly, no significant increase is seen, but if populations are separate, correlations are increased strongly when patches are smaller. In Figure 9C is depicted which network motifs are responsible for the increase of correlations. It can be observed that the difference in correlation is mainly due to differences in contributions of symmetric common input motifs 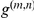 with 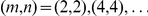, and to some extent of nearly symmetric ones (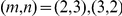). The reason is that if neurons of the same type receive common input, firing rates of their respective postsynaptic targets will be correlated. If their types differ, their targets receive correlated input of different signs, inducing negatively correlated rate fluctuations. Patchy output connections lead to an increased fraction of postsynaptic neurons of equal type if populations are spatially separated. In this case average correlations are increased. This effect is a direct consequence of the spatial organisation of neurons and connections. The same effect could however be achieved by assuming that single neurons preferentially connect to a specific neuron type.

A comparison of motif contributions to correlations, Figures 8C and 9C, shows that different architectures increase correlations via different motifs. Asymmetric motifs play a role in the correlation increase for hubs, but almost none for patchy networks.

## Discussion

We studied the relation between connectivity and spike train correlations in neural networks. Different rules for synaptic connectivity were compared with respect to their effects on the average and the distribution of correlations. Although we address specific neurobiological questions, one can speculate that our results may also be relevant in other areas where correlated activity fluctuations are of interest, such as in the study of gene-regulatory or metabolic networks.

### Hawkes processes as a model for neural activity

The framework of linearly interacting point processes in [38] provides a transparent description of equilibrium rates and correlations. It has been used previously to infer information about direct connectivity from correlations in small networks [44], as one amongst many other methods, see for example [49], [50] and references therein. Another application was the study of spike-time dependent plasticity [45], [51] and, in an extended framework, the description of spike train autocorrelations in mouse retinal ganglion cells [52]. An approach using linearised rate dynamics was applied to describe states of spontaneous activity and correlations in [53]. Correlations in populations of neurons have been studied in a rate model in [36] and in a point process framework in [37]. Hawkes' point process theory allows the treatment of correlations on the level of spike trains as well as the understanding of the relation of complex connectivity patterns to the statistics of pairwise correlations.

Although Hawkes' equations are an exact description of interacting point processes only for strictly excitatory interactions, numerical simulations show that predictions are accurate also for networks of excitatory and inhibitory neurons. Hence correlations can be calculated analytically even in effectively inhibitory networks in a wide range of parameters, as has already been proposed in [39]. One should note, however, that for networks with strong inhibition in combination with strong synaptic weights and low external input, low rates are not reproduced well.

The activity of cortical neurons is often characterised by low correlations [27], and can exhibit near-Poissonian spike train statistics [54] with a coefficient of variation near one. In theoretical work, similar activity has been found in balanced networks [41] in a certain input regime [40]. The level and time dependence of external input influences the general state of activity as well as pairwise correlations. In this study we are only concerned with an equilibrium resting state of a local network with asynchronous activity where external input is constant or unknown. We use Poisson processes as a phenomenological description for such a state and do not consider the biophysical mechanisms behind spiking activity, nor the reasons for asynchronous spiking on a network level. However, we found in simulations of networks of integrate and fire neurons of comparable connectivity parameters in an asynchronous-irregular state that correlations can be attributed to a large degree to linear effects of recurrent connectivity, although single neuron dynamics are nonlinear and spike train statistics are not ideally Poissonian (data not shown). Thus, although a linear treatment may seem like a strong simplification, this suggests that Hawkes' theory can be used as a generic linear approximation for the spike dynamics of complex networks of neurons. A similar point has been made in [53].

### Contribution of indirect synaptic interactions to correlations

We quantified correlations by integrated cross-correlation functions in a stationary state. The shape of the resulting correlation functions, which has been treated for example in [30], [37], [55], was not analysed. The advantage is that our results are independent of single neuron properties like the shape of the linear response kernel. Specific connectivity properties that can be described by a graph, as for example reviewed in [3], can be directly evaluated with respect to their effects on correlations.

In Hawkes' framework, taking into account contributions to pairwise correlations from direct interactions, indirect interactions, common input and interactions via longer paths is equivalent to a self-consistent description of correlations. This interpretation helps to derive analytical results for simple networks. Furthermore it allows an understanding of the way in which recurrent connectivity influences correlations via multiple feed-back and feed-forward channels. In particular, we showed why common input and direct input contributions are generally not sufficient to describe correlations quantitatively, even in a linear model. We showed that average correlations in networks with narrow degree distributions are largely independent of specific connectivity patterns. This agrees with results from a recent study [42], where conductance based neurons in two-dimensional networks with Gaussian connectivity were simulated. There, the degree of single neurons was kept fixed and population averaged correlations were shown to be invariant to different connectivity patterns. For net-inhibitory networks, indirect contributions to correlations effectively reduce average correlations. A similar effect has been described in [20] and in [36] for a rate model. In networks with strong recurrence, characterised by eigenvalues of the connectivity matrix close to one, correlation distributions are strongly influenced by higher-order contributions. In these networks broad distributions of correlations arise. In contrast, in very sparsely connected networks correlations depend mainly on direct connectivity.

Can we estimate the importance of recurrence from experimentally accessible parameters? In [56] the probability of a single extra input spike to generate an additional output spike, corresponding to 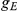, has been measured in rat barrel cortex in vivo as 0.019. Additionally, the number of connections made by each neuron was estimated to be about 1500. We now consider a local network with a fraction of inhibitory neurons of 20%. We assume an inhibitory synaptic weight 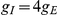 to balance the excitation, such that 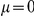. The estimated mean degree is consistent with many different topologies. Let us consider the case of a uniform random network of 15000 neurons with connection probability 0.1. For comparison we also look at a densely connected subnetwork of just 2500 neurons with a connection probability of 0.6. The first model results in a spectral radius 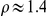 for the connectivity matrix 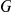, hence falling in the linearly unstable regime. In contrast, the second network displays a spectral radius slightly below one, which indicates linear stability. What can we conclude from this discussion? In the first place, this crude estimate of the spectral radius suggests that a value in the order of one is not an unrealistic assumption for real neural networks. This would call for a consistent treatment of long-range, higher-order interactions. This view is also supported by simulations of integrate and fire networks [31], which can yield similarly values for the spectral radius close to one. Our second example, although biologically less realistic, shows the range in which the spectral radius can vary, even if certain network parameters are kept fixed. This highlights the importance of the connectivity structure of local neural networks, as different network architectures can strongly affect the stability of a certain activity state.

### Effects of network architecture on correlations

We addressed ring networks with distance-dependent connection probability. Here, average correlations do not depend on the connectivity profile. However, for densely coupled neighbourhoods very broad correlation distributions can arise. A Mexican hat-like interaction has especially strong effects, since in that case higher-order contributions amplify correlations. This is not surprising since it is known that Mexican hat-like profiles can support large-scale activity patterns [57]. This implies that local inhibition increases network stability and leads to less extreme values for correlations. Distributions of correlations and distance dependence of correlations have been measured experimentally [20], [21], but they have not yet been related directly to anatomical connectivity parameters. In [19], the distance dependence of pairwise correlations as well as higher-order correlations has been measured experimentally. A generalisation of Hawkes' correlation equations in conjunction with the framework of cumulant-correlations discussed in [58] presents a promising route to study structure dependence also of higher-order correlations.

A generalisation to two-dimensional networks with distance dependent connectivity could be used to further investigate the relation between neural field models which describe large-scale dynamics [59]–[61] and random networks. However, the analysis using the full connectivity matrix allows to incorporate effects of random connectivity beyond the mean field limit. One example is that stability of networks is not only determined by mean recurrent input, but also by input variance.

Pairwise correlations affect activity in pooled spike trains [62]. We found that distance dependence of connectivity creates strongly coupled neighbourhoods and that population signals therefore depend on the connectivity statistics of the network. Such population signals could for example be related to local field potentials.

If the degree distribution is wide, networks can be constructed where connection probability depends on the out-degree of postsynaptic neurons. We considered networks where excitatory hubs, defined by a large out-degree, form a more or less densely connected subnetwork. Similar networks have been studied in [63]. In graph-theoretic terms, the connectivity between these hubs influences the assortativity of the network. A commonly used measure is the assortativity coefficient, which is the correlation coefficient between degrees of connected nodes. We calculated a generalised version for weighted networks, the weighted assortativity coefficient [64]. It can assume values between -1 and 1. Our networks have values between −0.22 and −0.05. Negative assortativity values are a consequence of the geometric degree distribution, but networks with more densely connected hubs have a higher coefficient. In our model, more assortative networks exhibit larger correlations than more disassortative ones. This illustrates how differences in higher-order statistics of connectivity can influence correlations, even if low order statistics do not differ.

In networks with patchy connections, an increase of correlations can be observed when populations of neurons are spatially non-homogeneous. Some information about how network architecture influences correlations can be obtained from examining contributions of individual motifs. In patchy networks mainly the contributions of symmetric motifs are higher, when excitatory and inhibitory neurons are separated, and therefore responsible for the correlation increase. In networks with hubs also asymmetric motifs play a role.

We found that fine-scale structure has important implications for the dynamics of neural networks. Under certain conditions, like narrow degree distributions, local connectivity has surprisingly little influence on global population averages. This suggests the use of mean-field models. On the other hand, broad degree distributions or the existence of connected hubs influence activity also on the population level. Such factors represent, in fact, major determinants of the activity state of a network and, therefore, should be explicitly considered in models of large scale network dynamics.

As considerable efforts are dedicated to the construction of detailed connection maps of brains on multiple scales, we believe that the analysis of the influence of detailed connectivity data, possibly with more refined models, has much to contribute to a better understanding of neural dynamics.

## Supporting Information

Text S1 Supporting information.
(PDF)
