# Supplementary material for: How Structure Determines Correlations in Neuronal Networks
Source: PLoS Comput Biol. 2011 May 19;7(5):e1002059. doi: 10.1371/journal.pcbi.1002059 (PMC3098224; doi:10.1371/journal.pcbi.1002059)
Supplement: Text S1 — Supporting information. (PDF) [file pcbi.1002059.s001.pdf]

Supporting Information

S1 Stability of the network

We want to determine the conditions for the stability of the time-independent solution of the equation

$$y(t) = y_0 + \int_{-\infty}^{\infty} G(t-\tau)s(\tau)d\tau = y_0 + (G * s)(t). \tag{1}$$

For simplicity we assume exponential interaction kernels, such that for non-zero elements $g_{ij}(t) = g_E\Theta(t)\exp(-t)$ for excitatory connections and $g_{ij}(t) = g_I\Theta(t)\exp(-t)$ for inhibitory ones. The elements of the integrated kernel matrix $\hat{G}(0)$ are then $\hat{g}_{ij}(0) \in \{g_E, g_I, 0\}$, depending on the connection type. Using the Fourier transform of the interaction kernel matrix, $\hat{G}(\omega) = \hat{G}(0)\frac{1}{1+i\omega}$, (1) can be transformed to

$$i\omega\hat{y}(\omega) = -\hat{y}(\omega) + \hat{G}(0)\hat{s}(\omega) + (1+i\omega)y_0\delta(\omega) \tag{2}$$

which formally becomes, after back-transformation, the differential equation

$$\frac{d}{dt}y(t) = -y(t) + \hat{G}(0)s(t) + y_0 + \frac{d}{dt}y_0. \tag{3}$$

Since y_0 is constant in time, $dy_0/dt = 0$. By definition, in an average over realisations $\langle s\rangle(t) = y(t)$, then

$$\frac{d}{dt}y = -[1-\hat{G}(0)]y + y_0. \tag{4}$$

This differential equation has a stable equilibrium solution if the real part of all eigenvalues of the matrix $[1-\hat{G}(0)]$ is larger than 0. This is exactly the case if, for all eigenvalues λ of G, $\Re(\lambda) < 1$. Since integrated interaction kernels correspond to postsynaptic spikes evoked by presynaptic input spikes, an intuitive interpretation is that no extra spike of any neuron evokes more than one additional spike in the network.

In order to interpret correlations as induced by recurrent input we use the series expansion $\sum_n G^n = (1-G)^{-1}$. The series converges only for matrices G with eigenvalues $|\lambda| < 1$. Although stable systems can exist with $|\lambda| > 1$ (one example are strongly inhibitory networks with $\Re(\lambda) < -1$) for this interpretation we have to restrict ourselves to systems where $|\lambda| < 1$ for all λ.

S2 Average correlations

General case

We want to show that the value of the average correlation in a regular network

$$\frac{1}{N^2}\sum_{ij}(c_{ij} - y_{ij}) = \sum_{(n,m)\neq(0,0)} g^{(n,m)} = \frac{\bar{y}}{N^2}\sum_{(n,m)\neq(0,0)}\sum_{ijk} g_{ik}^n g_{jk}^m \tag{5}$$

is determined by the out-degree parameters k_{ba}^{out}. Each term $\sum_{ijk} g_{ik}^n g_{jk}^m$ adds the weighted paths from all k to i in n and to j in m steps. We start with the case for $m = 1$ and $n = 1$. It needs to be treated separately from the ones for $n = 0$ or $m = 0$ because all neurons have only one type of output connection. The term $\sum_{ijk} g_{ik}g_{jk}$ counts the weights of common inputs in the network. There are N_{a_0} neurons of population $a_0 \in \{E, I\}$. Each causes common input into all possible pairs of neurons of the types a_1 and

b_1 of its postsynaptic populations. For each neuron there are $k^{out}_{a_1,a_0}$ and $k^{out}_{b_1,a_0}$ postsynaptic neurons of the respective population and the weight for each connection is g_{a_0}. Therefore

$$\sum_{ij}(GG^T)_{ij} = \sum_{a_0\in\{E,I\}} N_{a_0} g^2_{a_0} (\sum_{a_1\in\{E,I\},b_1\in\{E,I\}} k^{out}_{a_1a_0} k^{out}_{b_1a_0}) \tag{6}$$

Adding another step to the path corresponds to another factor G in (6). This leads to an increase in the number of possible paths. Each endpoint of one branch of the population a_1 can continue towards $k^{out}_{a_2a_1}$ neurons of a population a_2 with a weight g_{a_1}. Therefore, by induction, for $m, n > 0$,

$$g^{(n,m)} = \frac{\bar{y}}{N^2} \sum_{a_0,\dots,a_n} \sum_{b_1,\dots,b_m} N_{a_0} g^2_{a_0} g_{a_1} ... g_{a_{n-1}} g_{b_1} ... g_{b_{m-1}} k^{out}_{a_1a_0} ... k^{out}_{a_na_{n-1}} k^{out}_{b_1a_0} k^{out}_{b_2b_1} ... k^{out}_{b_mb_{m-1}} \tag{7}$$

For $n > 0, m = 0$ one obtains analogously

$$g^{(n,0)} = g^{(0,n)} = \frac{\bar{y}}{N^2} \sum_{ij} g^n_{ij} = \sum_{a_0,\dots,a_n} N_{a_0} g_{a_0} g_{a_1} ... g_{a_{n-1}} k^{out}_{a_1a_0} ... k^{out}_{a_na_{n-1}}. \tag{8}$$

These equations correspond to the fact that the total number of paths in a network does not depend on the specific connectivity, provided each neuron has the same number of output connections.

Special case

We treat the special case when there is a uniform connection probability p between all nodes

$$\frac{k^{out}_{ee}}{N_E} = \frac{k^{out}_{ei}}{N_E} = \frac{k^{out}_{ie}}{N_I} = \frac{k^{out}_{ii}}{N_I} = p. \tag{9}$$

For notational simplicity we use $k_{ab} = k^{out}_{ab}$ in this calculation. For the first order term, from (8) for $n = 1$ and $N = N_E + N_I$ one finds

$$g^{(1,0)} = \frac{\bar{y}}{N^2} \sum_{ij} g_{ij} = \frac{\bar{y}}{N^2} [g_E N_E (k_{EE} + k_{IE}) + g_I N_I (k_{EI} + k_{II})] = \bar{y}(g_E \frac{N_E}{N} p + g_I \frac{N_I}{N} p).$$

We define the average interaction between two nodes as

$$\mu \equiv \frac{g^{(1,0)}}{\bar{y}} = \frac{g^{(0,1)}}{\bar{y}} = g_E \frac{N_E}{N} p + g_I \frac{N_I}{N} p. \tag{10}$$

Similarly the common input term can be written as

$$g^{(1,1)} = \frac{\bar{y}}{N^2} \sum_{ijk} g_{ik} g_{jk} = \frac{\bar{y}}{N^2} [g^2_E N_E (k_{EE} + k_{IE})^2 + g^2_I N_I (k_{EI} + k_{II})^2] = \bar{y}(N_E g^2_E + N_I g^2_I) p^2,$$

and we define the average common input as

$$\eta = \frac{g^{(1,1)}}{\bar{y}} = (N_E g^2_E + N_I g^2_I) p^2. \tag{11}$$

Now we show that

$$g^{(0,n)} = g^{(n,0)} = \frac{\bar{y}}{N^2} \sum_{ij} g^n_{ij} = \mu^n N^{n-1} \bar{y}. \tag{12}$$

We start from (8):

$$
\begin{aligned}
\frac{\bar{y}}{N^2}\sum_{ij} g_{ij}^n &= \frac{\bar{y}}{N^2}\sum_{a_0,\ldots,a_n} N_{a_0} g_{a_0} g_{a_1} \ldots g_{a_{n-1}} k_{a_1 a_0} \ldots k_{a_n a_{n-1}} \\
&= \frac{\bar{y}}{N^2}\sum_{a_0,\ldots,a_{n-1}} N_{a_0} g_{a_0} g_{a_1} \ldots g_{a_{n-1}} \underbrace{k_{a_1 a_0}}_{pN_{a_1}} \ldots \underbrace{k_{a_{n-1} a_{n-2}}}_{pN_{a_{n-1}}} \underbrace{(k_{e,n-1} + k_{i,n-1})}_{pN} \\
&= \frac{\bar{y}}{N}\sum_{a_0,\ldots,a_{n-1}} N_{a_0} g_{a_0} g_{a_1} \ldots g_{a_{n-1}} pN_{a_1} \ldots pN_{a_{n-1}} p \\
&= \frac{\bar{y}}{N}(N_E p g_E + N_I p g_I)^n = \bar{y}(g_E \frac{N_E}{N} p + g_I \frac{N_I}{N} p) N^{n-1}. \qquad (13)
\end{aligned}
$$

In the same way, one can derive from (7) that for $n, m > 1$

$$
g^{(m,n)} = \bar{y}\eta\mu^{n+m-2} N^{n+m-2} \qquad (14)
$$

(12) and (14) can be derived in an alternativ way. If the average across the matrix $\frac{1}{N^2}\sum_{ij} \cdot_{ij}$ is replaced by an ensemble average, $\langle\cdot\rangle$, $\mu = \langle g_{ij}\rangle = \langle g^{(1,0)}\rangle/\bar{y}$ and $\eta = \langle (GG^T)_{ij}\rangle = \langle g^{(1,1)}\rangle/\bar{y}$. Then

$$
\langle (g^n)_{ij}\rangle = \sum_{k_1,k_2,\ldots} \langle g_{ik_1} g_{k_1 k_2} \ldots g_{k_{n-1} j}\rangle \approx \sum_{k_1,k_2,\ldots} \langle g_{ik_1}\rangle \langle g_{k_1 k_2}\rangle \ldots \langle g_{k_{n-1} j}\rangle = \mu^n N^{n-1}
$$

and, similarly

$$
\begin{aligned}
\langle (g^n (g^T)^m)_{ij}\rangle &\approx \sum_{k_1,k_2,\ldots,l_1,l_2\ldots} \langle g_{ik_1}\rangle \langle g_{k_1 k_2}\rangle \ldots \langle g_{k_{n-1},n}\rangle \langle (gg^T)_{k_n,l_m}\rangle \langle g_{l_{m-1} l_{m-2}}\rangle \ldots \langle g_{j l_1}\rangle \\
&= \eta\mu^{n+m-2} N^{n+m-2} \qquad (15)
\end{aligned}
$$

Here we assume that all terms and connections are independent from each other, which is approximately true only for large matrices and $n, m \ll N$.
We finally calculate

$$
\begin{aligned}
c = \frac{1}{N^2}\sum_{ij}(c_{ij} - y_{ij}) &= \sum_{(n,m)\neq(0,0)}^{\infty} g^{(n,m)} \\
&= \bar{y}(\sum_{n=1} \mu^n N^{n-1} + \sum_{m=1} \mu^m N^{m-1} + \sum_{n,m=1} \mu^{n+m-2} N^{n+m-2} \eta) \\
&= \bar{y}(2\sum_{r=1} \mu^r N^{r-1} + \sum_{r=1} (r-1)\mu^{r-2} N^{r-2} \eta) \\
&= \bar{y}(2\mu \sum_{r=0} (\mu N)^r + \eta \sum_{r=0} r(\mu N)^{r-1}) \\
&= \bar{y}(\frac{2\mu}{1 - N\mu} + \frac{\eta}{(1 - N\mu)^2}). \qquad (16)
\end{aligned}
$$

S3 Distance dependent correlations

For distance dependent correlations we proceed analogously as in (15). We replace the sample average by an ensemble average

$$
c(d) = \frac{1}{N}\sum_i c_{i,i+d} \mathrel{\widehat{=}} \langle c_{i,i+d}\rangle \qquad (17)
$$

Additionally we assume that rates are independent of neuron output

$$\langle c_{i,i+d}\rangle = \sum_k \langle b_{ik} b_{i+d,k} y_{kk}\rangle \approx \sum_k \langle b_{ik} b_{i+d,k}\rangle\langle y_{kk}\rangle = \bar{y}\sum_k \langle b_{ik} b_{i+d,k}\rangle \tag{18}$$

With the definition of

$$\langle g^{(m,n)}\rangle(d) \equiv \sum_k \bar{y}\langle g^n_{ik} g^m_{i+d,k}\rangle,$$

$$\langle c_{i,i+d}\rangle = \sum_k \bar{y}\langle b_{ik} b_{i+d,k}\rangle = \sum_{m,n}\langle g^{(m,n)}\rangle(d). \tag{19}$$

We can approximate the $\langle g^{(m,n)}\rangle(d)$ by

$$\begin{aligned}\langle g^{(m,n)}\rangle(d) &= \sum_{k_0} \bar{y}\langle g^n_{ik_0} g^m_{i+d,k_0}\rangle = \bar{y} \sum_{k_0,k_1,\ldots,k_{n-1},l_1,\ldots,l_{m-1}} \langle g_{ik_{n-1}} \cdots g_{k_1k_0} g_{l_1k_0} \cdots g_{i+d,l_{m-1}}\rangle \\ &\approx \bar{y} \sum_{k,k_1,\ldots,k_{n-1},l_1,\ldots,l_{m-1}} \langle g_{ik_{n-1}}\rangle \ldots \langle g_{k_1k} g_{l_1k}\rangle \ldots \langle g_{i+d,l_{m-1}}\rangle \\ &= \bar{y}[\mu * \ldots * \eta * \ldots * \mu](d) = \mu *^{n-1} \eta *^{m-1} \mu(d)\end{aligned}$$

where $*$ is a discrete convolution, $\mu(d) \equiv \langle g_{i,i+d}\rangle$ and $\eta(d) = \langle \sum_k g_{ik} g_{i+d,k}\rangle$. Both μ and η can easily be deduced from the connection rules in a ring, see main text. Note that $\eta(d) \neq \mu * \mu(d)$ because neurons make either excitatory or inhibitory connections.

Analogously one finds $\langle g^{(n,0)}\rangle(d) = \langle g^{(0,n)}\rangle(d) = \bar{y}\mu *^n \mu(d)$. A closed expression for the sum (19) can be derived after a discrete Fourier transformation

$$\hat{c}(k) = \sum_{d=0}^{N-1} c(d) \exp(-id2\pi k/N).$$

Using $\widehat{\mu * \mu} = \hat{\mu}\hat{\mu}$ one can proceed as in (16), since for example $\widehat{\langle g^{(m,n)}\rangle}(k) = \bar{y}\hat{\mu}^{n-1}\hat{\eta}\hat{\mu}^{m-1}$. An analogous calculation then returns

$$\hat{c} = \bar{y}(1 + \frac{2\hat{\mu}}{1-\hat{\mu}} + \frac{\hat{\eta}}{(1-\hat{\mu})^2}) \tag{20}$$

The additional summand 1 is due to the fact that we did not subtract the rate contribution to the autocorrelations here. Finally

$$c(d) = \frac{1}{N}\sum_{k=0}^{N-1} \hat{c}(k) \exp(ik2\pi d/N). \tag{21}$$
